# 3D network with channel excitation and knowledge distillation for action recognition

**DOI:** 10.3389/fnbot.2023.1050167

**Published:** 2023-03-23

**Authors:** Zhengping Hu, Jianzeng Mao, Jianxin Yao, Shuai Bi

**Affiliations:** ^1^School of Information Science and Engineering, Yanshan University, Qinhuangdao, China; ^2^Hebei Key Laboratory of Information Transmission and Signal Processing, Qinhuangdao, China

**Keywords:** action recognition, channel excitation, knowledge distillation, 3D convolution, deep learning

## Abstract

Modern action recognition techniques frequently employ two networks: the spatial stream, which accepts input from RGB frames, and the temporal stream, which accepts input from optical flow. Recent researches use 3D convolutional neural networks that employ spatiotemporal filters on both streams. Although mixing flow with RGB enhances performance, correct optical flow computation is expensive and adds delay to action recognition. In this study, we present a method for training a 3D CNN using RGB frames that replicates the motion stream and, as a result, does not require flow calculation during testing. To begin, in contrast to the SE block, we suggest a channel excitation module (CE module). Experiments have shown that the CE module can improve the feature extraction capabilities of a 3D network and that the effect is superior to the SE block. Second, for action recognition training, we adopt a linear mix of loss based on knowledge distillation and standard cross-entropy loss to effectively leverage appearance and motion information. The Intensified Motion RGB Stream is the stream trained with this combined loss (IMRS). IMRS surpasses RGB or Flow as a single stream; for example, HMDB51 achieves 73.5% accuracy, while RGB and Flow streams score 65.6% and 69.1% accuracy, respectively. Extensive experiments confirm the effectiveness of our proposed method. The comparison with other models proves that our model has good competitiveness in behavior recognition.

## 1. Introduction

With the advent of new, sophisticated deep learning architectures that are based on 3D Convolutional Neural Network variations, video processing has advanced dramatically in recent years (Diba et al., 2018; Feichtenhofer et al., 2019; Feichtenhofer, 2020; Zhu et al., 2020; Fayyaz et al., 2021). They have excelled at both the upstream and downstream tasks of video action recognition (Jiang et al., 2018; Xu et al., 2020; Zhao et al., 2020). However, it can be difficult and expensive to install these networks for inference tasks. Recent work treats recognition from motion as its objective, in which a “temporal stream” observes just a hand-designed motion representation as input, while another network, the “spatial stream,” observes the raw RGB video frames (Simonyan and Zisserman, 2014). When the spatial stream is a 3D Convolutional Neural Network, however, it has Spatio-temporal filters that respond to motion in the video. This, in theory, should allow the spatial stream to learn motion properties, a notion supported by the research (Tran et al., 2015; Lee et al., 2018). However, integrating a “temporal” 3D CNN that takes an explicit motion representation, often optical flow, as input yields significant accuracy gains (Carreira and Zisserman, 2017). The method of mixing 3D CNN-based RGB and Flow streams delivers better results, but it has considerable downsides. For starters, two-stream techniques necessitate explicit and precise optical flow extraction from RGB frames, which is computationally demanding. Second, the optical flow must be evaluated before the network's forward pass can be computed. Thus, two-stream techniques not only necessitate a large number of CPU resources but also result in excessive latency when identifying actions in an online context.

**Figure 1:**
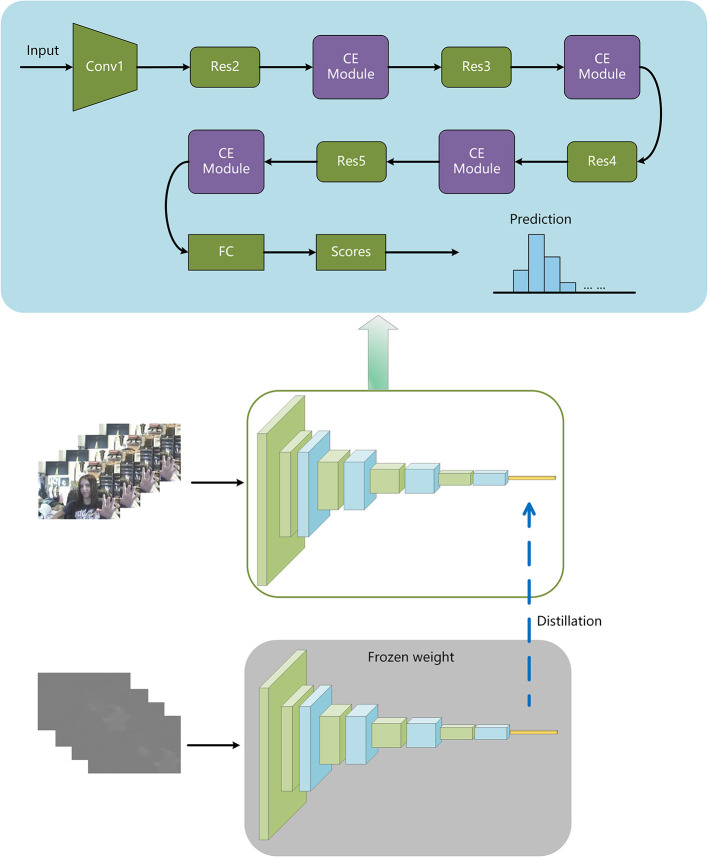
Training to make use of motion and appearance data. Initially, we use optical flow clips with cross-entropy loss to train the flow stream to identify activities and then freeze its weights. IMRS exploits both motion and appearance information by backpropagating the linear combination loss between features' overall network levels.

In this study, we present a unique learning strategy based on channel excitation and the distillation idea that avoids flow computation at test time while maintaining the performance of two-stream approaches. To begin, we train a 3D CNN with channel excitation on RGB input that hallucinates features from the Flow stream. More specifically, we minimize the difference between high-level features from the layer preceding the network's last fully-connected layer and motion stream features at the same level (see Figure 1). In other words, our stream is architecturally and input-wise comparable to the RGB stream, but it is trained using a different loss function called the linear combination loss function. We demonstrate that by utilizing this method, flow features can be extracted from RGB frames without the need for explicit optical flow computation during inference. This network is referred to as the Intensified Motion RGB Stream for convenience (IMRS). IMRS demonstrates that by precisely simulating the Flow stream, knowledge gathered from optical flow can be efficiently transferred to a stream with RGB inputs based on 3D Convolutions. More crucially, it indicates that at test time, flow computation can be avoided. Experiments show that a network trained using our innovative approach outperforms individual RGB and Flow streams. This demonstrates how IMRS effectively uses both appearance and motion information. Specifically, IMRS achieves 74.1% accuracy on HMDB51 (split-1), whereas RGB and Flow achieve 67.6 and 70.2% accuracy, respectively. The main contributions of this paper are summarized as follows:

1. A channel excitation module CE is presented to improve the effectiveness of video frame feature representation by strengthening the channel information interaction in a 3D network.
2. To improve the knowledge distillation effect between the teacher and student models, a linear combination loss function is developed.
3. With channel excitation and knowledge distillation, we design a network. Experiments suggest that it is more successful for action recognition issues, with higher accuracy on UCF101 and HMDB51.

## 2. Related work

Methods for recognizing video actions can be divided into two categories. To begin, there are 2D CNN techniques that use single-frame models to process each frame independently. Second, there are 3D CNN techniques, in which a model learns video-level information through the use of 3D filters. As we will see, both types of approaches frequently employ a two-stream approach, with one stream capturing features from appearance and the other capturing data from motion. Our research focuses on Two-Stream 3D CNNs.

### 2.1. 2D CNNs

Many ways take advantage of the power of single-image (2D) CNNs by applying a CNN to each video frame and pooling the predictions over time (Simonyan and Zisserman, 2014; Donahue et al., 2015). However, naive average pooling overlooks the video's temporal dynamism. Two-Stream Networks incorporate a second network termed the temporal stream to capture temporal information, which accepts a sequence of successive optical flow frames as input (Simonyan and Zisserman, 2014). The outputs of these networks are then integrated using late fusion or, in certain cases, early fusion, which involves allowing the early layers of the spatial and temporal streams to interact (Feichtenhofer et al., 2016). Other methods have used other approaches to include motion, such as modifying how characteristics are pooled across time with an LSTM or CRF (Donahue et al., 2015; Sigurdsson et al., 2017). These approaches have proven to be quite effective, especially when video data is scarce and training a 3D CNN is difficult. However, recent large-scale video dataset releases have accelerated progress in 3D CNNs (Zisserman et al., 2017).

### 2.2. 3D CNNs

By increasing the filters to three dimensions and applying them temporally, single-frame CNNs can be generalized to video (Ji et al., 2013). Because 3D CNNs have more parameters, they require more data to train. The earliest 3D CNNs were enabled by large-scale video datasets such as Sports-1M, but they were typically not significantly more accurate than 2D CNNs applied frame-by-frame, raising the question of whether 3D CNNs model motion (Karpathy et al., 2014). To compensate, many 3D CNN systems employ additional motion-incorporation algorithms. Motion is included in C3D utilizing Improved Dense Trajectory (IDT) features, resulting in a 5.2% gain in absolute accuracy on UCF-101(Wang and Schmid, 2013; Tran et al., 2015). Using a two-stream strategy in I3D, S3D-G, and R(2+1)D results in absolute improvements of 3.1, 2.5, and 1.1% on Kinetics, respectively (Carreira and Zisserman, 2017; Tran et al., 2018; Xie et al., 2018). These studies also show that optical flow input can significantly increase 3D CNN recognition ability.

### 2.3. Attention mechanism

The attention mechanism can direct the model's attention to key regions and enable the enhancement of critical features, hence enhancing recognition performance. SENet (Hu et al., 2018) uses the Squeeze and Excitation (SE) module to explicitly characterize channel dependency and improve channel characteristics. Woo et al. built an attention module (Convolutional Block Attention Module, CBAM) based on the channel excitation module to perform adaptive feature refinement on the input feature map (Woo et al., 2018). Based on a self-attention mechanism, Fu et al. suggested a dual-attention network to capture feature interdependence in the spatial and channel dimensions separately (Fu et al., 2019). Chi et al. offer a Cross-Modality Attention (CMA) algorithm that allows a two-stream network to acquire information from other modalities in a hierarchical fashion (Chi et al., 2019). In this paper, we propose a channel excitation module (CE) starting from the structure of SE Block, and verify the effectiveness of the module through experiments.

### 2.4. Distillation

The concept of distillation is central to our proposed learning strategy. Distillation was first proposed for knowledge transfer from a complicated to a simple model by using the complex model's class probabilities as a “soft goal” for the smaller one (Hinton et al., 2015). In a similar vein, our goal is to transmit knowledge from the motion stream to a network that simply accepts RGB input and does not do explicit flow computation. In our scenario, optical flow, along with RGB, is available for training, but only RGB is available during test time.

## 3. Methodology

### 3.1. Network architecture

Figure 1 depicts the network structure described in this article. The instructor model is one of the optical flow inputs, while the RGB input is the student model. Freeze the parameters of the teacher model after training it with the cross-entropy loss, and then train the student model. During the student model's training, the optical flow is fed into the teacher model with frozen parameters, and the RGB stream is fed into the student model. The combined loss function introduced in this research is used to calculate the loss of the output of the student model's fully connected layer and the output of the teacher model's fully connected layer. Backpropagation is used in the student model to update the parameters so that they can take advantage of motion and appearance information.

We employ 3DResNext101 (Hara et al., 2018) as the network backbone in this paper to extract deep features from input consecutive video frames. 3DResNext101 utilizes ResNet's repeated layer technique, which minimizes network complexity while boosting network width and depth to increase classification accuracy. Table 1 shows the network size after we introduced the CE module. *F* denotes the number of feature map channels, *N* is the number of residual blocks in each convolutional layer, and classes the number of action categories. The convolutional layer Conv1 is a 3D convolutional layer with a convolution kernel size of 7 × 7 × 7, 64 output channels, stride (1, 2, 2), and padding (3, 3, 3). The subsequent residual layers Res2-Res5 are stacked by residual blocks.

**Table 1:** Network size information.

**Layer**	***N***	***F***	**Output size**
Input	–	3 or 2	16 × 112 × 112
Conv1	–	64	16 × 56 × 56
Max pool	–	64	8 × 28 × 28
Res2	3	256	8 × 28 × 28
CE module	–	256	8 × 28 × 28
Res3	4	512	4 × 14 × 14
CE module	–	512	4 × 14 × 14
Res4	23	1,024	2 × 7 × 7
CE module	–	1,024	2 × 7 × 7
Res5	3	2,048	1 × 4 × 4
CE module	–	2,048	1 × 4 × 4
Avgpool	–	2,048	1 × 1 × 1
FC	–	–	1 × classes

### 3.2. Channel excitation module

We insert a CE module after each residual layer of the network, hoping to increase channel excitation, to improve the network's ability to pay attention to relevant information. SE Block, which adaptively calibrates channel feature responses by explicitly modeling channel interdependencies, won first place in the ILSVRC2017 classification (Hu et al., 2018). We propose a channel excitation module (CE Module) based on the structure of the SE Block and test its usefulness through experiments.

#### 3.2.1. Local cross-channel interaction

**Figure 2:**
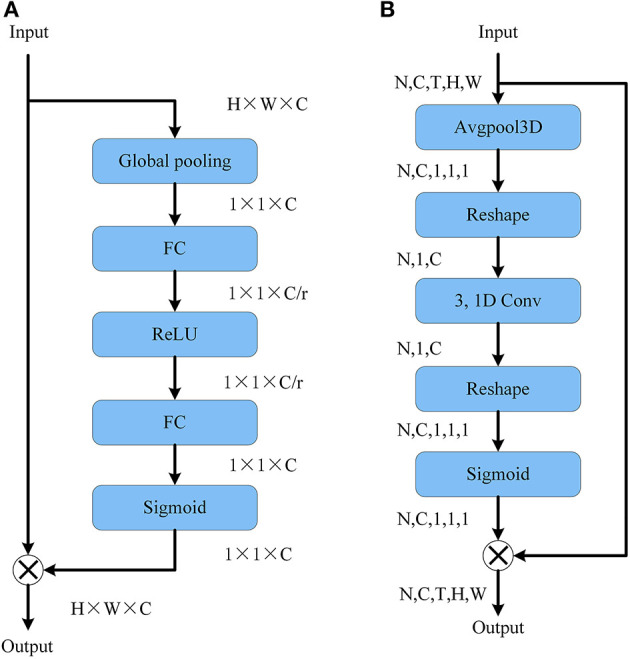
**(A)** SE block structure. **(B)** CE module structure.

SE Block reduces the dimension of the channel information using the squeeze operation, then utilizes the ReLU function to execute non-linear interaction on the squeezed channel information. As illustrated in Figure 2A, an expansion operation is added to the channel information after extrusion to restore the channel number before the extrusion operation. For a given feature *y*∈*R*^*C*^ without dimensionality reduction, the channel excitation can be learned by the following formula:


(1) $$\begin{array}{l} \eta =\sigma \left(W_{k}y\right), \end{array}$$

where η is the channel excitation coefficient, σ is the Sigmoid function, *W*_*k*_ is a parameter matrix of *k*×*C* dimension and its form is as follows:


(2) $$\begin{array}{l} W_{k}=\left[\begin{array}{c} \begin{array}{ccccc} w^{1,1} & \cdots & w^{1,k} & 0 & 0\\ 0 & w^{2,2} & \cdots & 0 & 0\\ \vdots & \vdots & \vdots & \ddots & \vdots \\ 0 & \cdots & 0 & \cdots & w^{C,C} \end{array} \end{array}\right]. \end{array}$$

Its meaning is: to calculate the weight *w*^*i*^ of the channel *y*_*i*_, only considering the interaction with its *k* neighbors, where *i* is the channel number, *j* is the number of the neighbors. That is


(3) $$\begin{array}{l} \eta_{i}=\sigma \left(\sum_{j=1}^{k}w_{i}^{j}y_{i}^{j}\right),\;y_{i}^{j}\in \Omega_{i}^{k}, \end{array}$$

where η_*i*_ is the channel excitation coefficient numbered *i* and $\Omega_{i}^{k}$ represents the set of *k* adjacent channels of the channel *y*_*i*_. One way is to have all channels share the same weights, i.e.,


(4) $$\begin{array}{l} \eta_{i}=\sigma \left(\sum_{j=1}^{k}w^{j}y_{i}^{j}\right),\;y_{i}^{j}\in \Omega_{i}^{k}. \end{array}$$

This method of parameter sharing can be implemented by a 1D convolution with a convolution kernel size of, i.e.,


(5) $$\begin{array}{l} \eta =\sigma \left(C1D_{k}\left(y\right)\right), \end{array}$$

where *C*1*D* represents 1D convolution. The method in formula (5) is implemented by the channel excitation module CE and only involves *k* parameters.

#### 3.2.2. Local cross-channel interaction scope

The extent of the interactions (i.e., kernel size of 1D convolutions) must be established for the CE module to correctly capture local cross-channel interactions. Because 3 × 3 is the most commonly used convolution kernel size in 2DCNNs and the number of parameters is limited, we select *k* = 3 as the default choice. Figure 2B depicts the construction of the CE module that we employed.

### 3.3. Knowledge distillation and loss function

Distillation was first proposed as a method for transferring knowledge from complex to simple models by employing complex model class probabilities as “soft targets” for smaller models (Hinton et al., 2015). Crasto et al. (2019) translates knowledge from motion flow to RGB input-only networks without explicitly computing optical flow. We train an RGB-enhanced flow model with high-level optical flow characteristics (Intensified Motion RGB Stream, IMRS) using knowledge distillation with a linear combination loss function, which requires just RGB inputs during testing.

In the field of image recognition, the cross-entropy loss is frequently employed as the classification model's loss function, and it takes the following form:


(6) $$Loss=CrossEntropy\left(s,\hat{y}\right),$$

where *s* is the class score predicted by the model and ŷ is the true class. The proposed teacher model in this paper only uses optical flow as input, and adopts cross-entropy loss for model training, i.e.,


(7) $$L_{Flow}=CrossEntropy\left(s_{Flow},\hat{y}\right),$$

where *L*_*Flow*_ is the teacher model classification loss and *s*_*Flow*_ is the class score predicted by the teacher model. A linear combination loss function of the following form is used to train the student model using only RGB input:


(8) $$\begin{array}{l} L=CrossEntropy\left(s_{RGB},\hat{y}\right)+\lambda_{1}\cdot \left\|fc_{RGB}-fc_{Flow}\right\|\\ +\lambda_{2}\cdot KL(P(fc_{RGB})||P(fc_{Flow})), \end{array}$$

where *s*_*RGB*_ is the class score predicted by the student model. CNN's first layer output represents low-level local information, while later layer outputs represent high-level global features (Zeiler and Fergus, 2014). *fc*_*RGB*_ and *fc*_*Flow*_ in formula (8) represent the high-level global features of the student model and the teacher model, λ_1_ is the scalar weight that adjusts the influence of the motion feature and λ_2_ is the scalar weight that adjusts the influence of the probability distribution of the motion feature. The values of λ_1_ and λ_2_ will be introduced in the experimental part. *KL*(*P*(*fc*_*RGB*_)||*P*(*fc*_*Flow*_)) denotes the relative entropy (Kullback-Leibler divergence) of the RGB model's high-level feature probability distribution and the FLOW model's high-level feature probability distribution. The formula for calculating relative entropy is as follows:


(9) $$\begin{array}{l} KL\left(P||Q\right)=\sum P\left(x\right)\frac{\log\left(P\left(x\right)\right)}{\log\left(Q\left(x\right)\right)}, \end{array}$$

where *P*(*x*) and *Q*(*x*) are the probability distributions of two discrete variables. In this paper *P*(*x*) and *Q*(*x*) are replaced by *P*(*fc*_*RGB*_) and *P*(*fc*_*Flow*_).

## 4. Experiments and evaluations

### 4.1. Datasets

**Figure 3:**
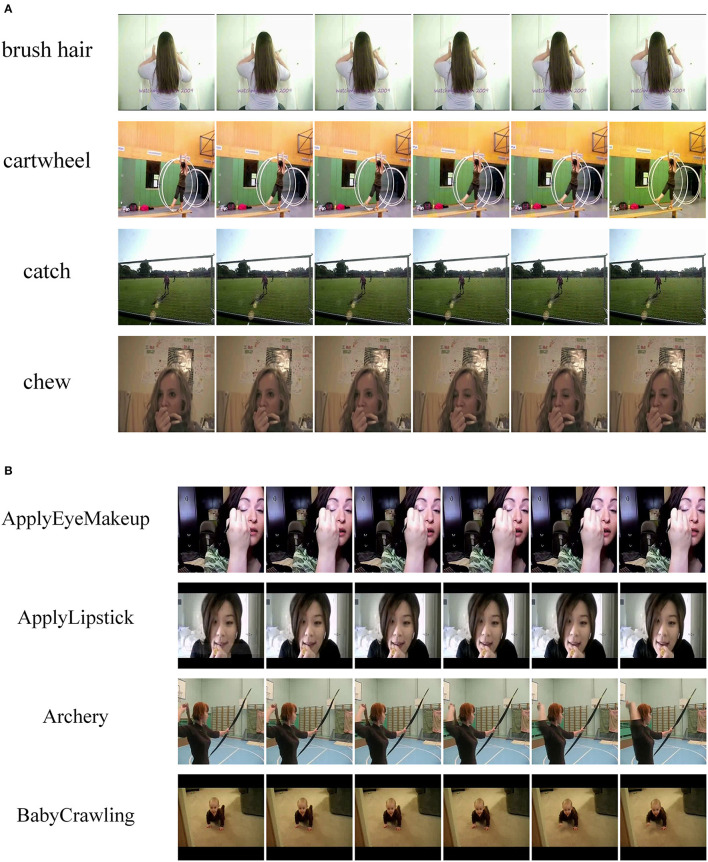
**(A)** HMDB51 dataset. **(B)** UCF101 dataset.

We concentrate on two popular action recognition benchmarks: HMDB51 (Kuehne et al., 2011) and UCF101 (Soomro et al., 2012). HMDB51 comprises 6,849 video clips divided into 51 activity categories. Human actions, face actions, and interactive activities are all featured in the videos. UCF101 comprises 13,320 video clips with an average duration of around 7 s and 101 action categories. Human-object interaction, human-human contact, human movement, sports, and musical instrument performance are some of the activity categories covered in videos. Figure 3 shows some of the data from the above two datasets. The two datasets mentioned here were trained and evaluated using the three officially provided splits, which means that there is no intersection between the training and evaluation data. To the best of our knowledge, the same works as the dataset used in our work are (Wang et al., 2016b; Crasto et al., 2019; Li et al., 2021). The first splits of HMDB51 and UCF101 are denoted as HMDB51-1 and UCF101-1, respectively.

### 4.2. Implementation details

The experiments in this paper generate models and conduct research in the Python3.6 environment using the Pytorch deep learning framework. All programs are executed on a server that has a V100 GPU.

#### 4.2.1. Data preprocessing

In this paper, the OpenCV toolkit in the Python environment is used to frame the original video dataset and extract the optical flow. In the process of video framing, all frames of the video data are extracted, and the image size of the video frame is adjusted to 256 × 256 pixels and saved in jpeg format. After the video frame is divided, the optical flow extraction operation is performed. In this paper, the TV-L1 (Zach et al., 2007) method is used to extract the optical flow, and the default parameter settings of OpenCV are used. We truncate the value of the optical flow file between −20 and 20 and map it to the (0,255) pixel range, saving it in jpeg format.

#### 4.2.2. Training

We use consecutive 16 frames as input during training after setting (Hara et al., 2018). A random cut to 112 × 112 size is performed on the input image, and a horizontal flip is randomly applied, which includes a random horizontal flip of the x-direction component for the optical flow input. Subtract the mean of the ActivityNet distribution for RGB input and 127.5 for FLOW input, assuming that the FLOW distribution is centered at 0. Following the settings of Hara et al. (2018), we adopt the SGD optimization method with a weight decay of 0.0005, a momentum of 0.9, and an initial learning rate of 0.1 for *ab initio* training. During the fine-tuning phase, we use a pre-trained model on the Kinetics400 dataset with a learning rate of 0.001, and when the performance no longer improves for 10 consecutive epochs, the learning rate becomes 0.1 times the previous one. The number of training cycles in this paper is 80 epochs, the batch size in the training phase is 32, and the batch size in the testing phase is 1.

### 4.3. Ablation experiments

#### 4.3.1. Impact of CE module

In this study, we investigate the effect of CE module position and number on model performance using RGB input on HMDB51-1. Table 2 shows that the ideal method for embedding the CE module into the network is to add a CE module to each Res2-Res5 residual layer, for a total of 4 CE modules added to the network.

**Table 2:** The influence of CE module position and number.

**Location**	**Number**	**HMDB51-1(%)**
Res_2_	1	65.9
Res_3_	1	65.8
Res_4_	1	67.0
Res_5_	1	66.2
Res_2 − 5_	4	**67.6**

The bold values represent the highest accuracy.

In this research, we investigate the effects of the SE block and the CE module on model performance using RGB input on UCF101-1 and HMDB51-1, respectively. It can be seen from Table 3 that the amount of model parameters hardly increases after the CE module is embedded in the network. Compared with adding the SE block to the network, the number of parameters is less and higher recognition accuracy is achieved.

**Table 3:** The effect of the CE module.

**Model**	**Params (M)**	**UCF101-1 (%)**	**HMDB51-1 (%)**
3DResNeXt101	48.34	90.7	63.8
3DResNeXt101+SE	49.04	91.0	65.4
3DResNeXt101+CE	48.34	**91.8**	**67.6**

The bold values represent the highest accuracy.

#### 4.3.2. The effect of **λ**_**1**_ and **λ**_**2**_

Regarding the linear combination loss function scalar weights λ_1_ and λ_2_ in formula (8), we conduct an experimental manual search on HMDB51-1. The experimental results are shown in Table 4. The optimal parameters obtained from the experiment are λ_1_ = 50, λ_2_ = 200.

**Table 4:** The effect of **λ**_**1**_ and **λ**_**2**_.

**λ_1_**	**λ_2_**	**HMDB51-1(%)**
1	1	73.1
10	10	73.3
50	50	72.1
50	100	73.3
50	200	**74.1**
50	300	73.0

The bold values represent the highest accuracy.

### 4.4. Results and discussion

**Figure 4:**
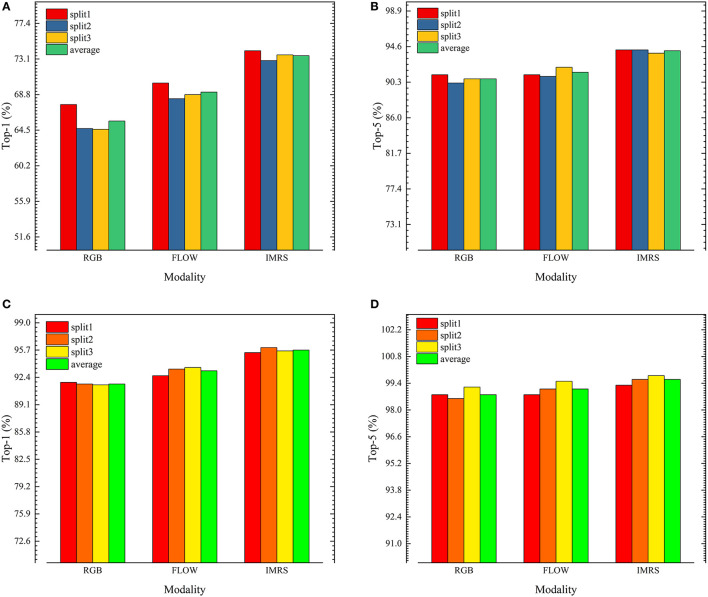
**(A)** HMDB51 accuracy top-1. **(B)** HMDB51 accuracy top-5. **(C)** UCF101 accuracy top-1. **(D)** UCF101 accuracy top-5.

Figure 4 depicts the accuracies of the single-stream 3DResNext101+CE model on the UCF101 and HMDB51 validation sets, with average accuracy over *Top-1* and *Top-5*. The overall performance of the dataset is calculated by averaging the dataset's three parts.

In terms of single-stream performance, the FLOW stream outperforms the RGB stream, demonstrating that motion information is more effective than appearance information for action recognition. The models all outperformed HMDB51 on UCF101, indicating that HMDB51 is more difficult to recognize actions than UCF101.

On both datasets, IMRS outperforms RGB and FLOW single-stream, indicating that the strategy of transferring motion flow information to appearance flow in the form of knowledge distillation is effective. On HMDB51-1, IMRS *Top-1* accuracy is 6.5 and 3.9% greater than pure RGB and FLOW streams, respectively. On UCF101-1, IMRS *Top-1* accuracy is 3.6 and 2.8% greater than pure RGB and FLOW streams, respectively.

### 4.5. Comparison with mainstream methods

Table 5 compares the model suggested in this paper to the industry standard approaches for behavior recognition. This paper's recognition effect on UCF101 and HMDB51 is the average of three splits. Our method outperforms the two-stream method in terms of recognition accuracy, but the number of parameters and computation rises due to the usage of 3D convolution.

**Table 5:** Compared with the mainstream methods.

**Method**	**Frame**	**Params (M)**	**FLOPs (G)**	**Pre train**	**UCF101 (%)**	**HMDB51 (%)**
Two-stream (Simonyan and Zisserman, 2014)	1+1	12	–	I	88.0	59.4
TSN(3 modalities) (Wang et al., 2016a)	3+3	10.4	16.4	I	94.2	69.4
CMA_*iter_1_*_R (Chi et al., 2019)	64	43.74	–	K	95.3	–
AMFNet-C (Liu and Ma, 2022)	6+5	–	–	I	95.9	71.2
RGB-I3D (Carreira and Zisserman, 2017)	64	12	108	I+K	95.6	74.8
T3D+TSN (Diba et al., 2017)	16	–	–	K	93.2	63.5
S3D-G (Xie et al., 2018)	64	11.56	71.38	I+K	96.8	75.9
3DResNeXt101 (Hara et al., 2018)	16	48.34	9.57	K	90.7	63.8
ECO.En (Zolfaghari et al., 2018)	{16,20,24,32}	150	267	K	94.8	72.4
MARS (Crasto et al., 2019)	16	95.23	18.03	K	94.6 (s1)	72.3 (s1)
3DCNN Ensemble+iDT (Huang et al., 2020)	36	1324.7	–	I	92.7	69.1
STDA-ResNeXt-101 (Li J. et al., 2020)	64	382	–	K	95.5	72.7
TSM (Lin et al., 2019)	8	24.3	33	I+K	95.9	73.5
STM (Jiang et al., 2019)	16	23.88	32.93	I+K	96.2	72.2
TEA (Li Y. et al., 2020)	16	–	70	I+K	96.9	73.3
CT-Net (Li et al., 2021)	16	–	145.5	I+K	96.2	73.2
IMRS (ours)	16	95.23	18.04	K	**95.7**	**73.5**

“S” stands for Sports-1M, “I” stands for ImageNet, “K” stands for Kinetics, “s1” stands for the first split and “–” means that the original text does not report this item. The bold values highlight the effect of our proposed method.

Compared with 3D CNN methods, our method outperforms some models, such as T3D+TSN (Diba et al., 2017), and ECO.En (Zolfaghari et al., 2018), 3DCNN Ensemble+iDT (Huang et al., 2020), STDA-ResNeXt-101 (Li J. et al., 2020). Compared with MARS (Crasto et al., 2019), the channel excitation module is introduced in the network structure, which brings a small number of parameters and a small amount of calculation. MARS only reported the accuracy on split1 for 16 frames input in the original text, and the average effect of this paper on the three splits of the dataset was higher than that of MARS. Among them, the performance of S3D-G (Xie et al., 2018) on the two datasets is higher than IMRS proposed in this paper. The main reason is that S3D-G is pre-trained on ImageNet+Kinetics and needs 64-frame input. In this paper, we just use 16 frames of input and solely employ Kinetics' pretrained parameters. On UCF101 and HMDB51, S3D-G is 1.1 and 2.4% higher than ours, respectively, but the computational cost of ours is much lower than S3D-G (18.04 vs. 71.38). On HMDB51, RGB-I3D (Carreira and Zisserman, 2017) is 1.3% higher than ours, but it is higher in terms of the number of input frames and computation.

Compared with the 2D CNN method, the method in this paper has an advantage in terms of computational complexity, and the recognition accuracy is less different from TSM (Lin et al., 2019). On HMDB51, our technique has a 1.3% greater recognition accuracy than STM (Jiang et al., 2019). On UCF101, our method's recognition accuracy is lower than that of 2D CNN, but it has an absolute advantage in terms of computational complexity. Based on these findings, we can conclude that our method has some advantages over conventional methods.

## 5. Conclusions and discussions

We present an action recognition network based on channel excitation and knowledge distillation in this work. A channel excitation module is employed by the network to improve channel information interaction and the network's capacity to extract relevant features. At the same time, the constitutes the linear combination loss function to train the teacher-student model to improve the student model's knowledge-learning ability in comparison to the teacher model. Furthermore, during student model inference, the network only takes RGB as input, which decreases model inference delay, and demonstrates good performance on behavior recognition tasks, which has reference relevance for the research of video behavior recognition algorithms. Future studies will concentrate on reducing the number of model parameters to boost recognition accuracy yet further. At the same time, we will focus on improving the training speed of the model and reducing the reasoning time.

In this work, we apply attention mechanism and knowledge distillation to the field of behavior recognition, and the experimental results show that these methods are simple and effective. We believe that this approach is not only suitable for solving action recognition problems, subsequent work could consider extending this approach to other fields, such as action prediction and anticipation (Dessalene et al., 2021), and robot self-learning (Yang et al., 2015). We will explore the application of this method to action prediction in the future.

## Data availability statement

Publicly available datasets were analyzed in this study. This data can be found here: https://www.crcv.ucf.edu/data/UCF101.php and https://serre-lab.clps.brown.edu/resource/hmdb-a-large-human-motion-database/.

## Author contributions

ZH: conceptualization and methodology. JM: writing and original draft preparation. JY: formal analysis. SB: resources and data curation. All authors contributed to the article and approved the submitted version.
